# Italian guidelines for primary headaches: 2012 revised version

**DOI:** 10.1007/s10194-012-0437-6

**Published:** 2012-05-12

**Authors:** Paola Sarchielli, Franco Granella, Maria Pia Prudenzano, Luigi Alberto Pini, Vincenzo Guidetti, Giorgio Bono, Lorenzo Pinessi, Massimo Alessandri, Fabio Antonaci, Marcello Fanciullacci, Anna Ferrari, Mario Guazzelli, Giuseppe Nappi, Grazia Sances, Giorgio Sandrini, Lidia Savi, Cristina Tassorelli, Giorgio Zanchin

**Affiliations:** 1 Headache Centre, Neurologic Clinic, University of Perugia, Perugia, Italy; 2 Neurologic Clinic, University of Parma, Parma, Italy; 3 Headache Centre, Neurologic Clinic “L. Amaducci”, University of Bari, Bari, Italy; 4 Headache Centre, University of Reggio Emilia and Modena, Modena, Italy; 5 Department of Child and Adolescent Neurology, Psychiatry and Rehabilitation, “Sapienza”, University of Rome, Rome, Italy; 6 Neurology Unit, Ospedale di Circolo e Fondazione Macchi, University of Insubria, Varese, Italy; 7 Neurologia II, Department of Neurology, Headache Centre, University of Turin, Turin, Italy; 8 Headache Centre, Department of Internal Medicine, Misericordia Hospital, Grosseto, Italy; 10 Florence, Italy; 11 Department of Psychiatry, Neurobiology, Pharmacology and Biotechnologies, University of Pisa, Pisa, Italy; 12 Headache Science Centre, C. Mondino National Institute of Neurology Foundation IRCCS, Pavia, Italy; 13 Department of Neurosciences, Headache Centre, University of Padua, Padua, Italy; 14 Department of Public Health, Neuroscience, Experimental and Forensic Medicine, University of Pavia, Pavia, Italy

**Keywords:** Guidelines, Primary headaches, Symptomatic and prophylactic treatment, Pharmacological and non pharmacological

## Abstract

The first edition of the Italian diagnostic and therapeutic guidelines for primary headaches in adults was published in J Headache Pain 2(Suppl. 1):105–190 (2001). Ten years later, the guideline committee of the Italian Society for the Study of Headaches (SISC) decided it was time to update therapeutic guidelines. A literature search was carried out on Medline database, and all articles on primary headache treatments in English, German, French and Italian published from February 2001 to December 2011 were taken into account. Only randomized controlled trials (RCT) and meta-analyses were analysed for each drug. If RCT were lacking, open studies and case series were also examined. According to the previous edition, four levels of recommendation were defined on the basis of levels of evidence, scientific strength of evidence and clinical effectiveness. Recommendations for symptomatic and prophylactic treatment of migraine and cluster headache were therefore revised with respect to previous 2001 guidelines and a section was dedicated to non-pharmacological treatment. This article reports a summary of the revised version published in extenso in an Italian version.

## Introduction and methodology

Ten years after the first edition (2001), the Italian Society for the Study of Headaches (SISC) decided to update the diagnostic and therapeutic guidelines for primary headaches in adults, not only including migraine, but also tension-type headache, trigeminal-autonomic cephalgias (TACs) and other primary headaches.

This concise version synthetically reports only treatment aspects (including non-pharmacological treatments and interventions), referring to the International Headache Society classification (ICHD-II, 2004) and its Appendix for diagnostic criteria. Therapeutic approach to other primary headaches has already been published by the members of Other Primary Headaches Subcommittee and therefore is not included in this updated version of Italian Primary Headaches Guidelines [2–5].

A literature search was performed on Medline database, considering all the articles on primary headache diagnosis and treatment published in English, German, French and Italian from February 2001 to December 2011. Only randomized controlled trials (RCT) and meta-analyses were analysed for each drug, if available. Lacking RCT, open studies and case series were also examined.

Four levels of recommendation were defined on the basis of the levels of evidence, the scientific strength of evidence and clinical effectiveness (Tables 1, 2, 3, 4).

**Table 1:** Levels of evidence

Level A: Two or more clinically controlled, randomize, double-blind studies carried out according to good clinical practice (GCP) versus placebo or versus an active drug for which there is proven evidence of efficacy
Level B: One clinically controlled study according to GCP or more than one controlled case–control study/ies or Cohort study/ies
Level C: Favourable judgement of two-thirds of the Ad Hoc Committee, historical controls, non-randomized studies, case reports

**Table 2:** Scientific strength of evidence

+++	The difference in the parameters of efficacy registered in studies compared with placebo or another active drug has a high level of significance (*p* < 0.01; *p* < 0.001; *p* < 0.0001). Adverse events are rare or occasional and not severe
++	The difference in the parameters of efficacy registered in studies reaches the minimum level of significance (*p* < 0.05) or the minimum clinically significant level (difference in the parameters <15 %)^a^
+	The difference in the efficacy parameters between the study drug and placebo or another active drug is not statistically significant
0	The drug is not efficacious or is characterized by severe adverse events

^a^Even drugs for which the difference in the efficacy parameters compared with placebo or another active drug is higher than the minimum level of statistical significance, but have frequent, yet no severe adverse events are included in this group

**Table 3:** Assessment of the clinical effectiveness of treatments

Symptomatic drugs	
+++	The majority (≥60 %) of the patients had partial or total relief of headache. More than 30 % of them were pain free
++	Many patients (from ≥40 to <60 %) had partial or total relief of headache, or 20–29 % of the patients were pain free
+	Some of the patients (from 20 to <40 %) had partial or total relief of headache. Up to 20 % were pain free
0	Less than 20 % of the treated patients received a clinical benefit
?	The members of the Ad Hoc Committee were unable to express any judgement on effectiveness based on their personal clinical impressions
Preventive drugs	
+++	The majority (≥50 %) of the patients experienced a reduction, of at least 50 %, in the frequency (and intensity) of attacks
++	Many patients (from ≥30 to <50 %) experienced a reduction, of at least 50 %, in the frequency (and intensity) of attacks
+	Some of the patients (from ≥20 to <30 %) experienced a reduction, of at least 50 %, in the frequency (and intensity) of attacks
0	Less than 20 % of the treated patients received a clinical benefit
?	The members of the Ad Hoc Committee were unable to express any judgement on effectiveness based on their personal clinical impressions

**Table 4:** Levels of recommendation for the pharmacological treatment of primary headaches

Level I	Drugs with high efficacy supported by statistically significant data (evidence of at least two controlled, randomized studies versus placebo or versus active drugs of proven efficacy) or very high clinical benefit for patients (clinical effectiveness +++) and with no severe adverse events
Level II	Drugs whose value of efficacy is statistically of lower significance compared to drugs of group I and with a less significant clinical benefit for patients (clinical effectiveness ++) and no severe adverse events
Level III	Drugs showing efficacy from a statistical point of view but not from a clinical point of view (contrasting results or evidence is not conclusive). The drugs belonging to this group were further subdivided into two subgroups: (a) Drugs with no severe adverse events (b) Unsafe drugs or with complex indications for use (e.g. special diets) or important pharmacological interactions
Level IV	Drugs of proven efficacy but with frequent and severe adverse events or drugs whose efficacy has not been proven from a clinical or statistical point of view (no difference with respect to placebo). Drugs with unknown clinical patient benefit or statistical significance of efficacy (data unavailable or insufficient)

Following the tradition, the management of primary headaches is divided into acute/symptomatic (to relieve headache attack) and preventive (to reduce frequency and intensity of headache attacks) treatment.

## Migraine

Symptomatic treatment of migraine attacks alone is recommended when attacks are non-disabling or, if disabling, they occur <4 days per month. Vice versa, a preventive treatment is recommended when disabling migraine attacks are ≥4 per month or, if <4 per month, in the case of poor response to symptomatic treatment. As in other primary headaches, a headache diary is a fundamental tool for monitoring attack frequency, duration and severity [6]. It is required when deciding the best treatment to suggest and for monitoring the effectiveness of symptomatic and prophylactic prescribed treatment.

### Acute attack treatment

1. A stratified approach, consisting in a different choice of initial treatment based on the severity of the attack (migraine-specific drugs, i.e. triptans, for moderate/severe attacks and non-specific drugs like analgesics and non-steroidal anti-inflammatory drugs (NSAIDs) for mild/moderate attacks) is recommended [7].
2. The most appropriate drug should be taken at the lowest useful dosage as early as possible after the attack begins.
3. As a rule, preparations with only one active principle should be preferred.
4. It is convenient to provide some alternatives for attacks of different severity.
5. Rescue drugs should be provided in case of first-choice medication failure.

### Symptomatic drugs

Drugs for migraine attacks include triptans, analgesics (NSAIDs), ergot derivatives and antiemetics.

#### Triptans

*Indications* They are indicated for the treatment of moderate or severe attacks (level of recommendation I). *Efficacy* RCT have demonstrated the efficacy of triptans, not only for headache but also for accompanying symptoms and functional disability [8–19] (Table 2). The consistency of efficacy of triptans in the treatment of multiple attacks and in long-term treatment (no development of tachyphylaxis) has also been shown [8, 9, 20]. They were also effective in menstrual-related migraine attacks [21]. Head-to-head studies did not establish the superiority of one triptan over the others [22]. Preference trials suggest that no ideal triptan exists for all patients, but the treatment must be tailored taking into account the characteristics of each patient and of the attacks [23–26]. Headache recurrence occurs in about 25–40 % of patients [8, 27, 28]. *Observations* When a triptan is administered early at the beginning of attack, it has a greater efficacy [29–31]. About 25–35 % of patients do not respond to a particular triptan, in which case other triptans can be tried [32–37]. In the case of an unsatisfactory response to a triptan or headache recurrence, a NSAID can be used [38]. Sumatriptan is available in all formulations (subcutaneous, tablet, nasal spray, suppository); subcutaneous sumatriptan is the most effective drug in the class [39–41]. Rizatriptan and zolmitriptan are available in rapid-dissolving formulations (RPD) which have an effectiveness similar to that of tablet formulations of the same drugs at the same dosages [42, 43]. Pharmacokinetics findings do not show higher blood levels reached at shorter times for RPD formulations. The latter can, however, be useful because they are easier to use without need of water, particularly when moderate or severe nausea is present. Naratriptan is not available in Italy. In some studies oral triptans at lower dosages have not demonstrated to be superior to some NSAIDs, simple or combination analgesics [21]. Oral triptan formulations are superior to oral ergotamine which has a low bioavailability (<1 %). Ergotamine and dihydroergotamine have an efficacy similar to that of triptans but induce more frequent adverse events [44–47]. The excessive use of triptans (≥10 days a month) exposes the patient to the risk of migraine chronification and should be avoided [48]. The concurrent use of triptans and NSAIDs seems to have a greater efficacy compared with that of triptans alone and is associated to lower headache recurrence [41–52]. *Side effects* are mostly mild-to-moderate, of short duration (10–15 min) and include triptan syndrome (chest and neck tightness, chest pain) in 4–5 % in patients treated with s.c. sumatriptan and 2–4 % with the oral formulation, fatigue, somnolence, dizziness and facial flush [6, 204–207]. Cardio- and cerebrovascular severe adverse events (myocardial infarction, ictus), without an established cause–effect relationship, were rarely reported. ECG modifications are also rarely reported [53]. Dystonic crises, akathisia, euphoria, can also rarely occur. *Contraindications* to triptans are uncontrolled blood hypertension, coronary artery disease, history of ischemic stroke, pheripheral artery disease, pregnancy and lactation and age >65 years.

#### Warnings

*Pregnancy and breastfeeding* From findings obtained by pregnancy registries, a greater number of preterm newborns or newborns with low birth-weight due to the use of sumatriptan during pregnancy have been described. In the case of repeated administration of sumatriptan in the first trimester there is no increased risk of newborn malformations but the sumatriptan use in the second and third trimester is associated with atonic uterus and bleeding >500 ml at delivery. Information on the safety of triptans during breastfeeding is limited but reassuring, because the minimal quantities secreted with milk are insufficient to induce adverse events to the child [54]. According to Italian Health Ministry Regulatory Agency the use of triptans is not recommended under 18 years of age with the exception of sumatriptan nasal spray 10 mg and zolmitriptan nasal spray 2.5 mg, which may be used in patients over 12 years of age [55, 56]. Also, according to this Agency, the use of triptans after 65 years of age is not recommended. They can be used only with a therapeutic plan approved by an Ethical Committee and with informed consent [57].

#### Pharmacological interactions

*Ergot derivates* A triptan can be used at least 24 h after ergot derivate administration. After taking a triptan it is necessary to wait at least 6 h before taking an ergot derivate.

*SSRI antidepressants* A serotoninergic syndrome can occur in the case of contemporary use of triptans and consists in motor incoordination, marked asthenia and hyperreflexia. *MAO-A inhibitors* They should be suspended at least 2 weeks before starting triptan treatment. *Propranolol* increases the concentration of rizatriptan. In the case of concomitant administration of propranolol, rizatriptan should be used at the dosage of 5 mg for the single attack, at the maximum daily dosage of 10 mg. After taking propranolol, it is necessary to wait at least 2 h before taking rizatriptan. *Drugs which are metabolized via CYP450* Eletriptan, rizatriptan and zolmitriptan may interact with drugs metabolized via CYP450 such as oral contraceptives and antimicotics. The clinical relevance of these observations needs to be clarified [58].

#### NSAIDs and analgesics

*Indications* They are indicated for the treatment of mild or moderate attacks or when triptans are contraindicated or ineffective [59, 60]. *Efficacy* The most consistent evidence of efficacy is available for *paracetamol, acetylsalicylic acid* (ASA), *lysine acetylsalicylate*, *naproxen sodium*, *ibuprofen*, *diclofenac sodium* and *potassium*, *metamizole* and *ketorolac*, whereas the evidence of efficacy for other NSAIDs is more limited [61–65]. Head-to-head studies have not shown a clear-cut superiority of a NSAID over another. Few studies have evaluated the efficacy of analgesics and NSAIDs on associated symptoms and functional disability. There is evidence only for ASA, salicylates, ibuprofen and diclofenac sodium. There are no studies supporting the consistency of efficacy and recurrence rates for the majority of analgesics/NSAIDs. The efficacy of ASA and other NSAIDs on migraine aura has never been tested.

*Observations* ASA is recommended in patients with cardio- and cerebrovascular comorbidities. *Paracetamol* is first-choice drug for migraine attacks during pregnancy. The excessive use of NSAIDs (≥15 days a month) should be avoided for the risk of migraine chronification. There is evidence of the use of ketorolac i.v. in the emergency department (ED), supporting its efficacy in the treatment of migraine attacks even if the results are less favourable than those obtained with prochlorperazine [66, 67]. In the same setting, ketorolac has been demonstrated to be more effective than sumatriptan nasal spray [68]. *Metamizole*, both by oral and intravenous route, has been demonstrated to be effective in the treatment of migraine attacks, but the risk of agranulocytosis and hypotension as relevant side effects should be considered [6]. *Side effects* consist mainly in gastrointestinal adverse events (from gastric pain to gastric or duodenal ulcer). The percentage of *adverse events* found in clinical trials concerning the use of NSAIDs for migraine attack are lower than those detected in studies regarding their daily use. These adverse events, occasional in migraine patients using sometimes NSAIDs, can occur with higher frequency in the case of daily or almost daily use by chronic migraine patients. *Contraindications* include severe renal and hepatic failure, hemorrhagic risk, gastric or duodenal ulcer.

*Warnings* Few NSAIDs can be used in patients under 14 years of age (ibuprofen, ketoprofen, morniflumate). NSAIDs should be administered with caution in elderly patients.

*Pharmacological interactions* Cumarol derivates or heparin (with the exception of those with low molecular weight): more risk of bleeding in the case of contemporary use with analgesics or NSAIDs. Alcohol should be avoided by concomitant use of analgesics or NSAIDs. Digoxin, barbiturates, lithium: NSAIDs increase their plasma concentration. Aldosterone, antagonists and potassium saving diuretics and antihypertensive drugs: NSAIDs reduce their efficacy.

#### Ergot derivatives

*Indications* Their use should be restricted to low frequency, severe attacks unresponsive to other drugs for their potential risk of abuse [69]. *Efficacy* Ergotamine tartrate with or without caffeine and dihydroergotamine have been demonstrated to be effective versus placebo or versus an active drug in reducing migraine headache [70, 71]. Ergotamine tartrate is not effective on nausea or vomiting; rather, because of the interaction with dopaminergic receptors, it may itself induce or increase nausea or vomiting accompanying the migraine attack [72]. Studies are lacking for its use for migraine aura. Ergotamine tartrate administration is associated with low incidence of recurrence (<30 %) [70]. Oral ergotamine is inferior to sumatriptan and eletriptan [44, 45].

*Observations Dihydroergotamine*, the drug of the class with the best risk–benefit ratio, is not available in Italy. Because nausea and vomiting may worsen due to the administration of ergot derivates, the contemporary administration of an antiemetic is generally indicated [72]. Caffeine doubles the rate of absorption of ergotamine and increases its peak blood concentration. This explains the development of combination formulations.

Patients who overuse ergotamine derivatives may develop rebound headaches. The abuse of ergot derivatives may induce an increase in the frequency of attacks and develop into a chronic headache. Therefore, these drugs are recommended for sporadic attacks and cannot be used for more than 10 days/month [48].

Major *side effects* Nausea, vomiting, diarrhoea and ergotism [73]. Ergotamine has a teratogenic effect [74]. *Contraindications* Cardiovascular and cerebrovascular diseases, uncontrolled blood hypertension, Raynaud disease, renal failure, pregnancy and lactation. *Pharmacological interactions* Triptans: an ergot derivate should not be administered within 6 h after the administration of a triptan. Beta-blockers: an increase in the risk of peripheral vasoconstriction has been observed in patients who also used beta-blockers. The majority of patients are able to tolerate such association; caution is necessary for particularly sensitive patients [58].

#### Combination analgesics

*Indications* They have the same indications of simple analgesics and NSAIDs. Few studies have been performed on these combination drugs [75]. *Efficacy* has been demonstrated only for the association with *acetylsalicylic acid, paracetamol* and *caffeine.* Recent trials have demonstrated a significant efficacy on migraine attacks of moderate intensity and moderate disability [76]. This association has been demonstrated to be effective in migraine attacks related to the menstrual cycle [76]. Recent data suggest that the effectiveness of the association of *indomethacin* + *caffeine* + *prochlorperazine* is similar to that of triptans, even though supporting studies are needed [77, 78]. Combination analgesics available in Italy include *acetylsalicylic acid* + *acethaminophen* + *propyhenazone, acetylsalicylic acid* + *acethaminophen* + *indomethacin* (with/without caffeine), and *acetaminophen* + *propyphenazone* and *acetaminophen* + *codeine.* Dosages of active substances in the combinations are different from those tested for migraine attacks. The efficacy of *acetylsalicylic acid* + *acethaminophen* + *propyphenazone and butalbital* + *propyphenazone* + *caffeine* has never been investigated in RCT for migraine*.*

*Observations* To avoid the risk of abuse, the use of combination analgesics should be limited to ≤10 days/month; abuse can lead to headache chronification [79–81]. *Side effects* and *contraindications* in combination analgesics are the same as those for each component. Caffeine may induce anxiety and insomnia.

#### Antiemetics

*Indications* Antiemetics are to be considered adjuvants, especially when nausea and vomiting are prominent [82]. *Efficacy* Most studies have concerned the association of antiemetics with analgesics and NSAIDs (naproxen, paracetamol, tolfenamic acid) or dihydroergotamine [72]. These associations have been proposed to improve the absorption of the symptomatic drugs and to act as adjuvants in reducing nausea or vomiting associated with the attacks. No RCT, however, clearly demonstrated a superiority of this association over NSAIDs alone. There is some evidence that suggests that the use of an antiemetic may improve the efficacy of a triptan [83]. *Metoclopramide, prochlorperazine* and *chlorpromazine* have also shown a modest antimigraine effect, besides a clear antiemetic effect [84, 85].

A modest antimigraine effect has been demonstrated for metoclopramide administered intramuscularly or intravenously [86].

Prochlorperazine or chlorpromazine administered intramuscularly or intravenously have been shown to be modestly effective in studies carried out in the ED. Oral prochlorperazine has also shown some partial efficacy [87, 88].

Dated findings on a limited number of patients support some efficacy of domperidone in preventing migraine attacks or reducing head pain intensity [326–328].

Intramuscular or intravenous formulations can be used in the treatment of attacks of severe intensity in which nausea and vomiting are prevailing and in the case in which other symptomatic drugs are contraindicated or sedation is needed. They can be considered as single drugs for the treatment of migraine in particular clinical settings (i.e. emergency department).

*Side effects* Metoclopramide may rarely induce dystonia, tardive dyskinesia and akathisia. The more frequent adverse events are somnolence and sedation. Rare adverse events are acute dystonic crises or akathisia and postural hypotension, particularly when an antihypertensive drug is coadministered.

The occurrence of adverse events due to phenothiazines is facilitated by alcohol or propranolol, which raises their plasma levels. Metoclopramide, prochlorperazine and chlorpromazine should not be coadministered with narcotics, sedatives, hypnotics and tranquilizers due to the synergic effects on the central nervous system. Prochlorperazine and chlorpromazine may lower the seizure threshold; they should be used with caution in patients with epilepsy. *Contraindications* Metoclopramide is contraindicated in patients affected by pheochromocytoma, epilepsy and in combination with neuroleptics such as phenothiazines, butyrophenones, MAOIs.

Antiemetics are not recommended in patients with prolactinoma. The use of metoclopramide, chlorpromazine and prochlorperazine must be limited only to cases of extreme necessity in pregnancy and during breast feeding [89].

*Pharmacological interactions* Anticholinergic drugs and antiacids may antagonize the effects of metoclopramide and domperidone on gastric motility.

#### Other drugs

*Simple or combination opioid analgesics* Controlled studies have demonstrated the association of paracetamol with codeine, doxilamine or buclizine to be no more effective than paracetamol alone [90]. In a more recent study the combination of paracetamol + codeine has been shown to be more efficacious than ASA [91]. The association of ASA with dextropropoxyphen and phenazone was not more effective than ergotamine [92].

Also, butorphanol (not available in Italy) by intramuscular route has not shown to be more effective than dihydroergotamine administered intravenously in association with metoclopramide.

There are no studies comparing butorphanol nasal spray with other non-opioid symptomatic antimigraine drugs [93, 94]. A double-blind study comparing the efficacy of ketorolac (60 mg) and meperidine (75 mg)/promethazine (25 mg), both administered by intramuscular route, did not show a statistically significant difference between the two drugs [95].

More recently, tramadol administered by intravenous route alone or in combination with paracetamol has been demonstrated effective in the acute treatment of migraine [96–98].

The Ad Hoc Committee has unanimously decided that this class of drugs does not represent a valid option for the symptomatic treatment of migraine attacks. This is due to the lack of data demonstrating their effectiveness compared with other symptomatic drugs and because of the potential risk of abuse and developing a chronic headache [99].

## Other drugs

### Barbiturates

There is no data supporting the efficacy of this class of drugs in the treatment of migraine crises [100]. Barbiturates may induce intoxication, addiction and dependence. High dosages may induce withdrawal syndrome after discontinuation. Their use should be avoided for the potential risk of abuse, rebound headache and chronification of migraine.

### Lidocaine

Limited evidence is available suggesting the effectiveness of this drug administered intravenously for the treatment of migraine attacks and in the chronic, refractory migraine unresponsive to other treatments, with or without symptomatic drug overuse [101].

Results of randomized, double-blind studies indicate a modest, but significant efficacy although with frequent and early recurrence [102, 103].

### Steroids

Available findings are conflicting and do not allow definitive conclusions to be drawn on their effectiveness in the treatment of migraine attacks, particularly in the case of refractory attacks and in reducing headache recurrence [104–108]. Steriods are indicated for the treatment of status migrainosus.

The group of experts recommends dexamethasone administered by intravenous route at a dosage of 10 mg or prednisone administered by oral route at a dosage of 50–100 mg in the treatment of status migrainosus, even though there are no consistent results from controlled trials versus placebo.

Limited findings are available for metilprednisolone [109, 110]. One study demonstrated the superiority of the association of dexamethasone and a triptan compared with triptan alone in the treatment of menstrual migraine attacks [111].

### Valproic acid

The drug administered by intravenous route at the dosage of 300–800 mg has been demonstrated to be effective in the treatment of migraine attacks. The promising results obtained need to be confirmed in double-blind, placebo-controlled studies involving a larger sample of patients [112].

### Gepants

They have been developed in recent years, and preclinical and clinical data suggest a role for calcitonin gene-related peptide (CGRP) in determining migraine attacks.

They include telcagepant (formerly MK-0974) whose efficacy has been shown in a study involving 500 migraine patients with good tolerability profile. The compound BI 44370 in a phase II study has shown its superiority compared with placebo at a dosage of 400 mg; phase III studies are still on going [113].

Further CGRP antagonists are currently being developed and a considerable expansion in this particular therapeutic area is expected. Currently these drugs are not available in the market and it is still too early to anticipate when they will be available for use in a clinical setting.

### Levels of recommendation

Table 5 shows symptomatic antimigraine drugs with levels of recommendation I and II.

**Table 5:** Drugs for the symptomatic treatment of migraine with a level of recommendation I and II

Drug	Dosage (mg)	Level of recommendation	Comments
5HT_1B/1D_ agonists			
Sumatriptan			
Subcutaneous	6		Rapid onset of action compared to the other formulations
Tablet	50–100	I	
Suppository	25		Useful when oral route is not possible due to nausea
Nasal spray	20		Useful when oral route is not possible due to nausea
Zolmitriptan			Rapid onset of action
Tablet	2.5		
Oral disintegrating tablet	2.5	I	
Nasal spray	2.5–5		
Rizatriptan			Rapid onset of action. The optimal dosage is 10 mg
Tablet	5–10	I	
Oral disintegrating tablet	10		Recommended dosage is 5 mg in patients treated with propranolol which increases the plasma concentration of rizatriptan
Eletriptan			
Tablet	20, 40	I	The optimal dosage is 40 mg (best efficacy/tolerability ratio) The dosage of 20 mg is recommended in the case of renal or liver failure
Almotriptan			
Tablet	12.5	I	Good tolerability profile
Frovatriptan			
Tablet	2.5	I	Long half-life, good tolerability profile
Ergot derivatives			
Ergotamine oral, rectal, subcutaneous	1–2	II	Indicated in the case of infrequent migraine attacks. Risk of abuse and headache chronification. An excessive use may cause ergotism
NSAIDs			
Acetylsalicylic acid (ASA) oral	500–1,000	I	Good efficacy/tolerability profile Gastrointestinal adverse events
Lisine acetylsalicylate oral	500–1,000	I	Good efficacy/tolerability profile Gastrointestinal adverse events
Lisine acetylsalycilate i.v.	1,000	I	To be used in a hospital setting. Risk of bleeding
Diclofenac–K+ oral	100	II	In the case of frequent migraine attacks risk of abuse and headache chronification
Diclofenac–Na+ i.m.	75	II	
Flurbiprofen oral	100–300	II	
Ibuprofen oral	400–1,200	I	
Ibuprofen oral	200	II	
Ketoprofen i.m.	100	II	
Ketorolac i.m. or i.v.	30–60	II	Clinical trials have been performed in particular settings (emergency departments)
Metamizole (dipirone) i.v. or oral	1,000	II	Potential risk of agranulocytosis >0.1 % and hypotension (i.v. formulation)
Naproxen oral	500–1,500	I	
Na + Naproxen oral	550–1,500	I	
Mefenamic acid per os	500	II	Effective in menstrual migraine attacks
Combination analgesics			
Paracetamol + acetyl salicylic + caffeine suppository	500 + 500 + 130		To be used for attacks of moderate intensity. Effective also in the treatment of menstrual migraine. In the case of frequent migraine attacks, risk of abuse and headache chronification
Indomethacin + prochlorperazine + caffeine oral	25 + 2 + 75	I	In the case of frequent migraine attacks, risk of abuse and headache chronification
Indomethacin + prochlorperazine + caffeine suppository	25–50 + 4–8 + 75–150	II	See above
Paracetamol + codeine per os	400–650 + 6–25	II	See above
Antiemetics			
Metoclopramide i.v.	0.1 /kg 1−3 times	II	To be used in a hospital setting

Table 6 includes symptomatic drugs with levels of recommendations III and IV.

**Table 6:** Drugs for symptomatic treatment of migraine with a level of recommendation III and IV

Drug	Route of administration	Dosage (mg)	Level of evidence	Scientific strength of evidence	Clinical effectiveness	Adverse events	Level of recommendation
NSAIDs							
Indomethacin	os	25–50	C	+	++	Frequent, not severe	III
	Rectal	50–100	C	+	++	Frequent, not severe	III
Nimesulide	os	100	C	+	+	Occasional, not severe	IV
Paracetamol	os	650–1,000	B	+	++	Rare, not severe	III
Piroxicam	Rapid dissolving formulation	40	B	++	+	Frequent, not severe	III
Ergot derivatives							
Ergotamine + caffeine	os, rectal	2 + 200	C	+	+	Frequent, not severe	III
Combination analgesics							
Butalbital + propyphenazone + caffeine	os	50 + 150 + 125; 175 + 25 + 75	C	0	+	Those of each active substance	IV
Antiemetics							
Metoclopramide	os	10	C	0	0/+	Infrequent	IV
Prochlorperazine	Rectal	20	B	++	+	Infrequent	III
Chlorpromazine	i.m.	0.1 /kg to 3 dosages	C	0	+	Occasional	IV
	i.v.	12.5–37.5	B	++	++	Slight to moderate	III
Domperidone	os	10	C	0	+	Rare	IV
Opioid analgesics							
Meperidine		50–100	B	++	++	Frequent, not severe	III
Tramadol		100	B	+	+	Occasional, not severe	III
Tramadol + paracetamol		37.5 + 325	B	+	+	Occasional, not severe	III
Other drugs							
Lidocaine	Intranasal	0.4 ml 4 % solution	B	++	+	Frequent, potentially severe	III
Prednisone	os	50–100	B	++	+	Frequent, potentially severe	III
Dexamethasone	i.v.	10	B	++	+	Frequent, potentially severe	III
Valproic acid		300–800	B	+	++	Frequent	III

### Preventive treatment

1. A good response to prophylactic treatment is obtained if there is at least a 50 % reduction in the frequency and severity of migraine attacks and a significant improvement in the quality of life is reached.
2. To minimize side effects and improve patient’s compliance, the most appropriate drug should be taken at the lowest dosage, preferentially as a monotherapy. Doses can be slowly increased until therapeutic goals are achieved without side effects.
3. Prophylactic treatment should be maintained for at least 3 months. Clinical benefit may take some time to be obtained.
4. Prophylactic drugs should be chosen based on patient’s comorbidities.
5. Particular attention should be devoted to drug–drug and drug–food interactions.
6. Most preventive drugs may have a teratogenic effect. Women should use a safe contraception.
7. Prophylactic treatment during pregnancy should be limited to special situations, and in these cases drugs with lowest risk for the foetus should be preferred.

### Preventive drugs

Preventive drugs include beta-blockers, calcium channel blockers, 5HT antagonists, antidepressants, antiepileptic drugs, angiotensin inhibitors, dihydroergotamine, botulinum toxin A and supplements [114, 115].

#### Beta-blockers

These drugs are to be preferred in the case of hypertension or tachycardia [116, 117].

Their efficacy as preventive drugs for migraine has been fortuitously demonstrated in migraine patients with concurrent hypertension. The efficacy of atenolol, nadolol, timolol, bisoprolol and nebivolol is supported by few controlled studies [118–124]. Even though prophylactic treatment is generally not advisable in pregnancy, propranolol may be used with relative safety [125]. The abrupt suspension of these drugs can induce an increase in the frequency of migraine attacks and an increase of blood pressure.

*Adverse events* include fatigue, depression exacerbation and nightmares, which are the most frequent, while asthma, orthostatic hypotension, impotence, hallucinations and weight gain occur less frequently.

*Contraindications* include congestive heart failure, atrio-ventricular block, peripheral artheriopathy, Raynaud syndrome, asthma, diabetes and depression.

*Calcium channel antagonists* are particularly recommended for patients with anxiety and insomnia [126].

*Efficacy* *Flunarizine* is the most used drug. *Cinnarizine* has a good antimigraine action although few studies have been carried out to investigate its efficacy in migraine [127, 128]. There are insufficient data supporting the efficacy of nimodipine and ciclandelate in migraine [129]. Therapeutic effects become evident only after some months of treatment. *Cinnarizine* induces the CNS side effects and accumulation phenomena less frequently.

*Side effects* of *flunarizine* are somnolence, asthenia, weight gain, depression and extrapyramidal symptoms in the long-term treatment and occur more frequently in elderly patients; c*innarizine* can induce somnolence and epigastric pain which can be avoided by taking the drug on a full stomach, weight gain, extrapyramidal symptoms in the long-term treatment and occur more frequently in elderly patients [130–132].

*Contraindications* include Parkinson’s disease or extrapyramidal disturbances, obesity, pregnancy and breast feeding.

#### Antidepressants: tricyclics

Among tricyclics, there are only two double-blind studies versus placebo supporting the efficacy of amitryptiline [133–135].

*Amitryptiline* is the first-choice drug particularly in patients with comorbid anxiety and depression or with concomitant tension-type headache.

Prophylactic effectiveness is obtained with doses lower than those used for depression (10–20 mg/day) [135].

The most frequent adverse events are antimuscarinic effects such as dry mouth, constipation and sedation. Often an increase in appetite (craving) with a consequent weight gain, or occasionally orthostatic hypotension and impotence, may occur [134].

*Contraindications* include cardiac arrhythmias, prostatic hypertrophy, glaucoma and epilepsy.

#### Serotonin norepinephrine reuptake inhibitors (SNRIs), selective serotonin reuptake inhibitors (SSRIs) and noradrenergic and specific serotonergic antidepressants (NaSSAs)

Promising data have been obtained for *venlafaxine* but RCT have some methodological limitations [136–138]. Although *paroxetine*, *escitalopram*, *fluvoxamine* and *sertraline* are sometimes used for migraine prophylaxis due to their good tolerability profile, available data are limited and contrasting [139–143]. There are no data available for *mirtazapine*. There is also some positive evidence for fluoxetine [144, 145].

Venlafaxine can be useful in patients with depression and concomitant anxiety. Fluoxetine can induce insomnia, fatigue, tremor and epigastric pain. SSRI can interfere with 5HT_1_ agonists.

#### Antiepileptic drugs

*Sodium valproate* and *topiramate* are first-choice drugs in the treatment of high-frequency migraine attacks, chronic migraine, with and without symptomatic drug overuse and in the case of comorbid epilepsy [146, 147].

Sodium valproate and topiramate are effective in both migraine with and without aura and in chronic forms. Their long-term efficacy has been shown [148–153]. The dosages useful for the prophylactic treatment of migraine are lower than those used for epilepsy. *Gabapentin* has a good tolerability profile [154]. In open studies, *lamotrigine* (50–200 mg/day) has been shown to be effective in the treatment of high-frequency migraine attack with aura [155–157]. It is ineffective in migraine without aura [158]. Promising data have been obtained for *zonisamide*, *levetiracetam*, *pregabalin* and need to be confirmed.

*Contraindications* are hepatitis, pancreatitis, thrombocytemia for sodium valproate; liver and kidney insufficiency, kidney stones, glaucoma for topiramate; hypersensitivity to the drug for gabapentin and lamotrigine. All antiepileptics are contraindicated during pregnancy due to their teratogenic effect [159].

Adverse events caused by sodium valproate include asthenia, dizziness, tremor, alopecia, weight gain, menstrual disorders, hepatopathy and pancreatitis; for topiramate, asthenia, memory problems, anomia, weight loss, paresthesia, dysgeusia and depression; rare adverse events include metabolic acidosis, kidney stones, psychosis and narrow-angle glaucoma; for gabapentin asthenia, somnolence, ataxia, diplopia and constipation; for lamotrigine: skin rashes (which may be warning signs of Stevens–Johnson syndrome; to reduce this risk a slow titration is recommended), somnolence, gastroenteric disturbances and ataxia [160].

#### 5HT-antagonists

5HT-antagonists are the oldest drugs available for migraine prophylaxis and include *methysergide*, a semisynthetic derivative of *ergometrine*, which is not available in Italy and *pizotifen*, a serotonin antagonist with modest antihistaminic and cholinergic effects, which is a second-choice drug due to its side effects [161].

There are a few dated clinical studies supporting the efficacy of pizotifen [162–165]. This drug has a long half-life (about 23 h) and can be used in a unique dose (0.5–1.5 mg), for cycles of 3 months.

*Contraindications* include glaucoma, arrhythmias, urinary retention and obesity.

The most frequent *side effects* are somnolence, increase in appetite, weight gain, xerosthomia and constipation [166].

#### Angiotensin inhibitors

*Indications* *Lisinopril* and *candesartan* are second-choice drugs to be considered for patients with a concomitant hypertension [167–169]. There is only one positive RCT for both drugs [170, 171].

*Contraindications* are angioedema, bilateral stenosis of renal artery for lisinopril; hypersensitivity to sulphonamides, hypokaliemia, hypercalcemia, liver and kidney insufficiency and gout for candesartan.

Adverse events for lisinopril are asthenia, hypotension, dry cough, hyperkalemia, gastrointestinal disturbances and impotence; for candesartan asthenia, dizziness, tachycardia and hyperuricemy.

*Other drugs* Two ergot derivatives, controlled release *dihydroergotamine* 10 mg/day and *dihydroergocryptine* 20 mg/day (not available in Italy), can be taken into account as second-line treatments [172, 173]. *Riboflavin* at high doses (400 mg) showed a certain efficacy in preventing migraine, with few side effects (moderate abdominal pain, diarrhoea), in one RCT [174]. This drug is available as a galenic preparation in Italy. Among herbal remedies, butterbur root extract (*Petasites hybridus*), at the dosage of 150 mg/day, proved to be effective in two RCTs, while another herbal drug, *Tanacetum parthenium*, studied in several RCTs, gave more conflicting results [175, 176].

One controlled study versus placebo demonstrated the efficacy of *Coenzyme Q10* (100 mg × 3 per day) [177]. The superiority of *tiotic acid*, a drug which also increases brain energetic metabolism, has also been shown compared with placebo [178]. Conflicting findings have been obtained for pidolate magnesium (400–600 mg/day) [179, 180]. The use of non-chelated formulations determines diarrhoea at clinically efficacious doses. It can be used in women with menstrual migraine and premenstrual syndrome, but a precise administration schedule has not been established (646–648). OnabotulinumtoxinA showed some efficacy in various open studies in migraine patients, but contradictory results emerged in double-blind controlled studies versus placebo concerning patients with episodic migraine [181]. These studies used different protocols, individualized or standardized, different inoculation sites and groups with different frequency of attacks (see chronic migraine).

*Menstrual migraine* In pure menstrual migraine or menstrually related migraine, a short-term prophylactic therapy around menses can be tried if menstrual cycles are regular and migraine attacks are predictable [182]. Two different strategies may be followed: (1) administration of symptomatic drugs, in particular triptans, on a regular basis instead of on demand; (2) administration of *estrogens*, to avoid the premenstrual *oestrogen* fall that is thought to be a main cause of menstrual migraine. In the first case, *frovatriptan* (5 mg/day) or *zolmitriptan* (5–7.5 mg/day) may be administered [183–185]. Positive results have also been obtained in one RCT for sumatriptan 100 mg and for *naproxen sodium* (1,100 mg/day) [186]. The administration should be limited to the period −2 +4/5 days from menstrual onset. If the second option is chosen, *estradiol gel* (1.5 mg/day) or *transdermal estradiol patch* (100 μg) are probably the best choices [187, 188].

*Chronic migraine* Only recently some RCTs have been carried out in patients affected by the most disabling form of migraine, i.e. chronic migraine. Two drugs have so far showed some evidence of efficacy: *topiramate* and *onabotulinumtoxinA*. Topiramate (100 mg/day) has been partially effective also in patients with medication overuse; onabotulinumtoxinA (injection every 12 weeks) has given a statistically significant clinical benefit [189–191].

Data supporting a significant efficacy of onabotulinumtoxinA have been obtained in patients with chronic migraine with or without symptomatic drug overuse [192–194].

### Levels of recommendation

Table 7 shows prophylactic drugs with levels of recommendation I and II. Table 8 includes preventive drugs with levels of recommendation III and IV.

**Table 7:** Drugs for the preventive treatment of migraine with a level of recommendation I and II

Drug (by oral route)	Daily dosage (mg)	Level of recommendation	Comments
Beta-blockers			
Propranolol	80–240	I	Useful in patients with hypertension, anxiety and panic disorders. It can exacerbate depression. Do not use with ergotamine. Increase doses gradually. Particularly useful in patients with essential tremor. Most frequent adverse events are fatigue, mood disorders, nightmares. Other side effects are bradicardia, orthostatic hypotension, impotence, hallucinations, weight gain
Metoprolol	50–200	I	Same indications and side effects as for propranolol, excluding essential tremor
Atenolol	100	I	Same indications and side effects as for propranolol, excluding essential tremor
Bisoprolol	5–10	II	Same indications and side effects as for propranolol, excluding essential tremor
Nadolol	40–240	II	
Calcium channel blockers			
Flunarizine	5–10	I	Use administration schedules with periodic suspensions (i.e. 5 days/week or 3 weeks/month), to avoid the accumulation of the drug Most frequent side effects are weight gain, sedation and depression. Extrapyramidal symptoms may be observed in elderly patients. The recommended dose to reduce adverse events is 5 mg
Cinnarizine	75–150	II	Most frequent side effects are weight gain and drowsiness
Antidepressants tricyclic			
Amitriptyline	10–75	I	Dosages tested in clinical trials, the majority of them dated, are in general higher than those usually used in clinical practice for prophylactic treatment of migraine A progressive increase in doses is recommended until maintenance doses are reached in order to reduce adverse events Most frequent side effects are drowsiness, weight gain and anticholinergic symptoms. Particularly useful in patients with depression, concurrent migraine and tension-type headache. Higher doses should be used in patients with comorbid depression
Antiepileptic drugs			
Sodium valproate	500–1,500	I	Controlled release formulations are available with a better tolerability profile. Recommended for patients with prolonged or atypical migraine aura. Not recommended in patients with liver disease and haemorrhagic diathesis. A progressive increase in doses is recommended. Frequent adverse events include nausea, asthenia, somnolence. Other side effects include weight gain, hair loss and tremor. Teratogenic potential
Topiramate	50–100	I	Gradual increase of dosage is recommended. Frequent, not serious adverse events include paresthesiae, memory and concentration disturbances, nausea, weight loss and drowsiness. Rare serious adverse events include kidney stones, narrow-angle glaucoma
Gabapentin	900–2,400	II	Recommended for elderly patients. Well tolerated
5HT-antagonists			
Pizotifen	1.5	II	Frequent adverse events include weight gain and somnolence
Other drugs			
Dihydroergotamine	10	II	Do not use within 6 h after triptan administration. Useful for intermittent or short-term prophylaxis. Withdrawal could be associated with rebound headache
Dihydroergocriptine	20	II	Mild side effects. Withdrawal could be associated with rebound headache
Onabotulinum toxin type A	155–195 U^a^	IV (episodic migraine) I (chronic migraine)	The majority of controlled studies have not provided conclusive results in episodic migraine It is effective in chronic migraine. Costs are comparable to topiramate 100 mg for a period of treatment of 3 months and lower than topiramate for a period of 4 months

^a^Dosage referred to each inoculation

**Table 8:** Drugs for the preventive treatment of migraine with a level of recommendation III and IV

Drug	Route of administration	Daily dosage (mg)	Level of evidence	Scientific strength of evidence	Clinical effectiveness	Adverse events	Level of recommendation
SNRI and SSRI							
Fluoxetine	os	10–40	B	+	+	Frequent, not severe	III
Venlafaxine	os	75–150	B	+	+	Occasional, not severe	III
Angiotensin inhibitors							
Lisinopril	os	5–20	B	+	+	Occasional, not severe	III
Candesartan		16	B	++	+	Rare, not severe	III
Antiepileptic drugs							
Lamotrigin	os	50–200	B	++	+++ (only migraine with aura)	Occasional, not severe	III
5HT_1_ antagonists							
Methysergide		2–8	A	+++	++	Potentially severe	III^a^
Other drugs							
Ribloflavin		400	B	++	+	Rare, not severe	III^b^
Magnesium		400–600	B	++	+	Rare, not severe	III^b^
*Petasites hybridus*		100–150	B	++	+	Rare, not severe	III^c^
*Tanacetum parthenium*		18.75	B	+	0/+	Rare, not severe	III^c^
Thiotic acid		600	B	+	?	Rare, not severe	III^c^

^a^Not available in Italy

^b^Not available in Italy at the recommended dosages

^c^Available in Italy as a herbal product or as an over-the-counter product

## Tension-type headache (TTH)

### Acute attack treatment

Just like in migraine, the treatment of the acute episode is necessary. Patients should record attack frequency, duration and severity in a headache diary to monitor the disease course and the effectiveness of therapy.

#### General considerations

1. The most appropriate drug should be taken at the first symptoms and at the lowest dosage useful to obtain a complete resolution of the crises and as early as possible.
2. Formulations containing solely one active drug should be preferred.
3. The patient should fill in a headache diary to evaluate headache recurrence, treatment efficacy and potential side effects.
4. The choice of the symptomatic drug/s should be based on the careful and critical consideration of clinical data, the mechanism of action and side effects of the drugs.

### Symptomatic drugs

Drugs for TTH attacks include NSAIDs, simple analgesics, antiemetics and other drugs.

#### NSAIDs

Scientific evidence supports the efficacy of *acetylsalicylic acid* (ASA), *diclofenac*, *ibuprofen*, *ketoprofen*, *metamizol* (dipyrone) and *naproxen* [195–205].

ASA, ketoprofen and naproxen are first-choice drugs. ASA is especially useful in the case of comorbid cerebrovascular or cardiovascular disease, but is to be avoided in patients with gastric disease. Low doses of NSAIDs are usually sufficient to obtain a therapeutic result. *Ibuprofen* is reported to have minor gastric adverse effects. Comparative studies were not able to demonstrate the superiority of one drug because the comparisons were not based upon equivalent doses of the different molecules [196, 198–201, 204, 205]. The assumption of NSAIDs or simple analgesics for 15 days or more per month, for >3 months, can induce a chronification of headache and a chronic medication-overuse headache.

*For contraindications, drug interactions and side effects,* see the paragraph on migraine.

#### Simple analgesics

*Paracetamol* has been tested in TTH patients showing good efficacy and tolerability [196, 200, 204]. It is a first-choice drug both in pregnancy and in patients with gastric disease but it should be used with caution in subjects with liver disease.

*Combination analgesics* with *caffeine*, *codeine* and *butalbital* share indications with simple analgesics and are more effective than the latter. The combination drugs with caffeine (*paracetamol* + *caffeine*, *paracetamol* + *ASA* + *caffeine* and *ibuprofen* + *caffeine*) are classified at recommendation level I [206–210]. Also, the association of *indometacin* + *prochlorperazine* + *caffeine* was demonstrated to be effective in one study [207].

An excessive and frequent use of combination analgesics (for 10 days a month or more) should be avoided because of the high risk of drug abuse, headache chronification and drug-induced headache. *Paracetamol* + *codeine* (500 + 30 mg) and *butalbital* + *propyphenazone* + *caffeine* (50–150 + 125–175 + 25–75 mg) are recommended at the level III because of the addiction potential [211, 212]. Patients taking opiates or barbiturates should be informed about the risks deriving from an abrupt discontinuation and undergo a hospitalized drug discontinuation schedule. Combination analgesics share the same contraindications, drug interactions and side effects of the single components (for details see “Migraine”).

*Antiemetics* are generally not necessary in the usual management of TTH acute therapy. Intravenous metoclopramide, however, was found to be effective in the emergency treatment of TTH episodes [213]. Also, intravenous administration of chlorpromazine was effective in the ER TTH management [214].

*Complementary or alternative drugs* Promising results have been obtained with a topical preparation of peppermint oil 10 g and ethanol (90 %) to 100 [215, 216]. Tiger balm has also shown to have a modest but significant effect in inducing headache relief [217].

### Preventive treatment

#### General considerations

1. A preventive therapy is recommended in the case of headache-related disability for ≥4 days/month or poor response to symptomatic treatment even if the headache frequency is lower.
2. Treatment is considered effective if it reduces attack frequency and/or severity by at least 50 %.
3. The identification of trigger factors and their elimination, when possible, contribute to reduce attack frequency [218].
4. Comorbid diseases play a main role in the choice of therapy (e.g., the use of amitriptyline is contraindicated in prostatic hypertrophy and glaucoma).
5. Particular attention should be devoted to drug interactions.
6. Preventive therapy should always be based on a single drug which should be titrated to the lowest effective and well tolerated dose.
7. No recommendations are available concerning the best duration of treatment.
8. Patients should be involved in the choice of treatment and it is advisable to use a limited number of administrations (compliance is in fact inversely proportional to the number of administrations).
9. Patients should also be informed on how and when drugs should be taken, on their potential side effects and their efficacy. Patients should be advised to record their attacks in the headache diary to verify frequency and duration of headache, functional impairment, number of symptomatic drugs taken, efficacy of prevention treatment and possible side effects.

### Preventive drugs

Drugs for prevention therapy of TTH include antidepressants (tricyclics, SSRI, other antidepressants), muscle relaxants, benzodiazepines and other drugs.

#### Antidepressants

Among *tricyclics* *amitriptyline* is a first-choice drug. It is also recommended in case of comorbidity with anxiety, insomnia, depression or migraine [219, 220]. *Amitriptyline* is utilized at much lower dose for TTH than that necessary to obtain an antidepressive effect. A slow titration of the drug is recommended to increase its tolerability and avoid adverse events.

*Contraindications* include cardiac arrhythmias, prostatic hypertrophy, glaucoma and epilepsy. It should be used with caution in the elderly because of its anticholinergic action. For side effects see paragraph on migraine. Also, *clomipramine* (150 mg/die) and *desipramine* (75 mg/die) may be useful [221, 222].

*SSRI* are less effective than amitriptyline but better tolerated [223, 224]. *Fluvoxamine* was the most studied molecule in this class. Also *fluoxetin* and *paroxetin* showed some evidence of efficacy.

#### Other antidepressants

*Mirtazapine*, a specific noradrenergic and serotoninergic antidepressant, is another first-choice drug which is especially indicated in the case of comorbid anxiety, insomnia and depression [225, 226]. It should be taken at low initial doses because it can induce sedation and sleepiness. Promising results come from *venlafaxin*, a noradrenalin and serotonin selective reuptake inhibitor (SNRI), which is able to reduce headache frequency independently of the association with anxiety or depression [227, 228]. *Maprotiline* and *mianserine* are other antidepressants that have shown to be effective in TTH preventive therapy [229, 230]. The latter was compared to both *clomipramine* and *fluvoxamine* with similar efficacy [221]. No conclusive results have been obtained for *nefazodone* [231] and *ritanserine* [232, 233], not available in Italy; sulpiride has been used at the dosage of 30 mg/day and has been shown to be more effective than *paroxetine* [234].

#### Muscle relaxants

One RCT supports the use of *tizanidine* in chronic headache including TTH. The drug was slowly titrated over 4 weeks to 24 mg or the maximum tolerated dose showing moderate adverse effects (somnolence, dizziness, dry mouth, asthenia) [235, 236]. In one study *cyclobenzaprine* has demonstrated to be effective in the improvement or complete relief of headache in half of 20 TTH patients, whereas only partial relief was obtained in one-third of patients treated with placebo [237].

#### Benzodiazepines

In dated studies *diazepam* (5 mg/day) seemed to be more effective than placebo in reducing headache frequency [238, 239]. It can be useful in the case of comorbid anxiety. Also, *alprazolam* (0.75 mg/day) has been demonstrated to be effective in TTH preventive therapy with a lower level of rating (level of recommendation III) [240].

#### Other drugs

Positive results have been obtained with *topiramate* (25–100 mg/die), which was tested at the initial dose of 25 mg/die titrated to 100 mg/die in chronic TTH patients in a recent open study [241]. *Buspirone,* a non-benzodiazepine anxyolitic drug, has been compared to amitriptyline, showing moderate results in a small sample of patients [220]. Further investigation is needed (level of recommendation III). *L*-*5*-*hydroxytryptophan* at the daily dose of 300 mg showed mild efficacy in the prophylaxis of chronic TTH in one RCT (level of recommendation III) [242]. Conflicting results have been found with *onabotulinumtoxinA* which may be attributed, at least in part, to variability of doses, study protocols, inoculation sites and headache frequency of enrolled patients. Further studies are needed (level of recommendation IV) [243, 244].

### Levels of recommendation

Table 9 shows symptomatic drugs for TTH with levels of recommendation I and II. Table 10 includes symptomatic drugs with level of recommendation III.

**Table 9:** Drugs for the symptomatic treatment of tension-type headache with a level of recommendation I and II

Drug	Dosage (mg)	Level of recommendation	Comments
Analgesics and NSAIDs			
Acetylsalicylic acid oral	500–1,000	I	Good efficacy and tolerability profile. Not recommended in pregnancy and in gastric disease
Diclofenac–K+ oral	12.5–50	II	
Ibuprofen oral	400–800	II	Reduced gastric damage
Ketoprofen oral	50–100	II	
Lumiracoxib	200–400	II	
Metamizol (dipyrone) oral	500–1,000	II	Potential risk of agranulocytosis >0.1 % and of hypotension
Metamizol (dipyrone) intravenous	1,000	II	Tested to treat TTH in the emergency room. Potential risk of agranulocytosis >0.1 % and of hypotension
Naproxen oral	275–550	I	
Paracetamol oral	500–1,000	I	Use with caution in patients with epatic failure
Combination analgesics			
Ibuprofen + caffeine oral	400 + 200	II	Risk of abuse and headache chronification with frequent use
Indometacin + prochlorperazine + caffeine oral	25 + 2 + 75	II	See above
Paracetamol + caffeine oral	500–1,000 + 30–130	I	See above
Paracetamol + acetyilsalicylic acid + caffeine oral	200–1,000 + 500 + 30–50	I	See above

**Table 10:** Drugs for the symptomatic treatment of tension-type headache with a level of recommendation III

Drug	Dosage (mg)	Level of evidence	Scientific strength of evidence	Clinical effectiveness	Adverse events
Combination analgesics					
Paracetamol + codeine oral	500 + 30	B	++	++	Occasional, not severe
Butalbital + propyphenazone + caffeine oral	50–150 + 125–175 + 25–75	B	++	++	Occasional, not severe
Antiemetics					
Metoclopramide intravenous	10	B	+	+	Moderate, not severe
Chlorpromazine intravenous	10	B	++	++	Frequent
Complementary alternative drugs					
Peppermint with ethanol (90 %) to 100 topic	10	B	++	++	Not recorded
Tiger balm topic	Not defined	B	++	+	Not recorded

In Table 11 preventive drugs for TTH with levels of recommendation I and II are listed. Table 12 reports preventive drugs with level of recommendation III.

**Table 11:** Drugs for the preventive treatment of tension-type headache with a level of recommendation I and II

Drug	Dosage (mg)	Level of evidence	Scientific strength of evidence	Clinical effectiveness	Adverse events	Level of recommendation	Comments
Antidepressants							
Amitriptyline	25–75	A	+++	+++	Frequent, not severe	I	Useful in patients with comorbid anxiety, depression, insomnia. Contraindicated in the case of glaucoma and prostatic hypertrophy
Clomipramine	10–150	B	++	++	Frequent, not severe	II	
Fluvoxamine	50–100	B	++	++	Frequent, not severe	II	
Maprotilin	75	B	+++	++	Frequent, not severe	II	
Mianserin	30–60	A	++	+	Frequent, not severe	II	
Mirtazapine	15–30	A	+++	+++	Frequent, not severe	I	Particularly indicated in patients with anxiety, depression, insomnia. It may induce somnolence
Venlafaxine	75–150	B	++	++	Frequent, not severe	II	
Muscle relaxants							
Tizanidine oral	3–12	B	+++	+++	Frequent, not severe	II	Especially useful in the case of pericranial muscle tenderness
Benzodiazepines							
Diazepam	5	B	++	++	Occasional, not severe	II	
Other drugs							
Topiramate oral	25–100	C	+++	+++	Frequent, not severe. Rarely severe	II

**Table 12:** Drugs for the preventive treatment of tension-type headache with a level of recommendation III

Drug	Dosage (mg)	Level of evidence	Scientific strength of evidence	Clinical effectiveness	Adverse events
Antidepressants					
Desipramine	75	C	+	++	Frequent, not severe
Fluoxetine	20	C	+	++	Frequent, not severe
Paroxetine	20–30	B	+	+	Frequent, not severe
Nefazodone	100–450	C	++	++	Frequent, not severe
Ritanserin	10	B	+	+	Frequent, not severe
Sulpiride	30	B	+	++	Frequent, not severe
Muscle relaxants					
Cyclobenzaprine oral	10	B	++	++	Frequent, not severe
Benzodiazepines					
Alprazolam oral	0.75	B	+	+	Occasional, not severe
Other drugs					
Buspirone	30	C	+	+	Frequent, not severe
L-5-hydroxytryptophan oral	300	B	+	+	Occasional, not severe

None of the drugs evaluated by the group of experts were included at level of recommendation IV

## Cluster headache (CH)

Treatment of CH is a difficult challenge and needs the active participation of the patients, who should be reassured on the benign nature of his/her headache. They should be informed on the drugs available for preventing or interrupting attacks and that at present there are no treatments able to prevent active periods and modify the natural history of the disease. CH patients should be advised on factors that may precipitate attacks during an active phase, in particular, alcoholic beverages that should therefore be avoided.

### Acute attack treatment

Treatment of CH attack should not be delayed. Drugs should be promptly bioavailable and this can be attained if they are administered by parenteral or subcutaneous route [245, 246].

The objectives of a correct and effective symptomatic treatment are (1) to treat the attack when it starts; (2) to obtain pain relief as soon as possible (possibly within 15 min from the administration of the drug); (3) to limit to a minimum the adverse events [247].

### Symptomatic drugs

#### Sumatriptan

Its efficacy has been demonstrated for the subcutaneous (s.c.) and intranasal formulations. *Sumatriptan* *s.c.* (6 mg) has been shown to be effective both on pain and associated symptoms [248–250]. When administered for long periods, it maintains its efficacy without tachyphylaxis and with a good safety profile [251, 252]. The efficacy of *sumatriptan nasal spray* (20 mg in a nostril) has been shown within 30 min after administration in a double-blind randomized study [253]. The greater latency of action suggests its use for attacks lasting at least 45 min. Sumatriptan nasal spray does not have the indication for cluster headache in Italy. The most common adverse events are reactions in the site of injection, dizziness, paraesthesia, sensation of cold or warm and irritation of the nostril for the intranasal formulation. In 90 % of cases they are mild or moderate [254].

#### Zolmitriptan

Its efficacy has been demonstrated for the oral and intranasal formulations. *Oral formulation* (5 and 10 mg) has been demonstrated to be effective at 30 min (reduction of 2 points of pain intensity in a 5-point scale) in one RCT [255]. In Italy the only dosage available is 5 mg (to be reached only once within 24 h if needed). *Zolmitriptan* *nasal spray* (5 and 10 mg) has also been shown to induce CH relief in two RCTs, with a higher efficacy for the 10-mg formulation and with few adverse events for both dosages [256–258].

A Cochrane analysis of six randomized studies, controlled versus placebo, demonstrated that sumatriptan and zolmitriptan are superior to placebo [254]. Adverse events of triptans include paraesthesia, asthenia, nausea, dizziness and irritation of the nostril for the intranasal formulation.

#### Oxygen by inhalation

The efficacy of *oxygen inhalation* at the flow of 7 l/min for 15 min has been shown in dated open studies and in a more recent controlled crossover study versus room air [259]. In a further randomized study using an oxygen mask and oxygen flow of 12 l/min a complete remission of cluster headache attacks both in the episodic and chronic forms was obtained within 15 min in 78 % of cases versus 20 % for room air [260]. In the case of no response to usual recommended flow it may be increased to 14–15 l/min [261].

#### Ergotamine derivatives

Dated studies have shown the efficacy of *ergotamine tartrate* 250 μg i.m. (only 1 study vs. placebo), *ergotamine tartrate* (1 mg) plus *caffeine* (100 or 200 mg) tablets (in a study also in association with *bellafoline* 100 mg) and *ergotamine tartrate* (2 mg) plus *caffeine* (100 mg) and *dihydroergotamine nasal spray* for the acute treatment both in patients with episodic and chronic forms [262–266].

#### Anaesthetics

Contrasting results have been obtained for *lidocaine* intranasal (4 %) in open studies [101, 267, 268]. Better results were achieved in a study using 10 % lidocaine solution with respect to saline [269].

The application of a solution of *cocaine* 10 % in both nostrils has been shown to block the attack in a controlled study versus placebo (saline solution) in patients with episodic and chronic cluster headache [269, 270]. No significant adverse events were recorded with the exception of a mild state of arousal in a patient who had abused the drug. Cocaine is not available in Italy as medication.

#### Somatostatin and somatostatin analogues

Two randomized controlled trials are available, one for somatostatin i.v. (25 μg in 50 ml saline) and one for octreotide i.v. (an analogue with longer half-life: 1.5 h, 100 μg in 1 ml vehicle) demonstrating a significant reduction of pain in 20 and 30 min. respectively [271, 272]. Most common side effects are nausea, diarrhoea and meteorism.

### Preventive treatment

Preventive treatment is a fundamental part of the management of active CH, which cannot be obtained only with the acute treatment due to the high frequency, suddenness and shortness of the attacks. A symptomatic treatment alone should be limited, in the episodic form, to active phases of short duration (mini cluster). The objectives of the preventive treatment are (1) the rapid disappearance of attacks and the resolution of active periods; (2) the reduction of frequency, intensity and duration of attacks [245, 246, 273].

The effectiveness of preventive treatment can be evaluated with certainty only in the chronic form, because in the episodic form there is always the doubt that the active period runs out spontaneously and not because of the treatment.

1. Preventive treatment must start early in the active phase.
2. Treatment must continue for at least 2 weeks after disappearance of attacks.
3. Treatment must be suspended gradually.
4. If the attacks reappear, dosages must be increased to therapeutic levels.
5. Treatment must be re-started at the onset of a subsequent active period.
6. The choice of the drug depends on different factors: age and lifestyle of the patient (avoid alcohol intake during the cluster period); expected duration of the cluster period; type of CH (episodic or chronic); response to previous treatments; reported side effects; contraindications to recommended drugs; comorbid diseases.
7. Polytherapy, tested in a few trials, is indicated only in patients resistant to monotherapy or patients who do not tolerate recommended drugs at optimal dosage.

### Preventive drugs

#### Verapamil

In a double-blind controlled study versus placebo this drug at the dosage of 120 mg × 3 per day has demonstrated to be effective in patients with episodic CH. In these patients it is indicated as first-choice drug [274]. In the chronic form, according to two open studies and one head-to-head study versus lithium carbonate, it was effective in 50–55 % of the patients [275–277]. Dosages used were higher than those used for the episodic form (up to 960–1,200 mg). In the comparison study verapamil was effective more rapidly with fewer side effects [276]. The most relevant adverse events include arrhythmia (19 % of cases) and bradycardia (36 %); ECG monitoring of patients is recommended to avoid an atrioventricular block and symptomatic bradycardia [274, 278, 279]. In Italy, verapamil is not indicated for cluster headache.

#### Lithium salts

Results are available from some dated open studies and two RCTs demonstrating the effectiveness of this drug in the preventive treatment of chronic CH. In the two RCTs, dosages were 300 mg × 3 per day and 800 mg/day, respectively [277, 280, 281]. Blood lithium levels should range from 0.4 to 0.6 mmol/l. The most frequent adverse events include tremor, gastrointestinal disturbances, dizziness and polyuria [281, 282].

#### Steroids

In a retrospective study, *prednisone* (10–80 mg/day) induced a significant reduction (72 % of cases) or a complete remission (58 % of cases) of attacks within 3–10 days in a small sample of CH patients with episodic or chronic form, with the best results obtained for dosages ≥40 mg [283]. When dosage was gradually reduced, (<20 mg) the attacks reappeared. The drug is recommended for short-term usage (i.e., at the start of other recommended preventive treatments).

*Methylprednisolone* i.v. (250 mg in 100 ml saline) followed by *prednisone* per os (10 mg/day) induced a further benefit in patients treated with optimal doses of *verapamil* [284]. The i.v. administration of methylprednisolone (30 mg/kg in 500 ml of saline in 3 h) induced the interruption of the cluster period for 2–3 days, with a subsequent prompt reappearance of attacks [285]. No significant side effects were observed for both drugs at the used dosages.

#### Serotonin antagonists

*Methysergide* has been tested only in dated open studies which demonstrated an efficacy in 76–77 % of cases [286, 287]. The recommended dose is 8 mg (although the dosage of 16 mg has also been tested). The drug should be started with the dose of 2 mg and should be increased gradually every 3–7 days. Side effects occur in 20–45 % of patients. The most frequent include nausea, dizziness, stomach pain, restlessness, somnolence and cramps. The drug should not be used for more than 6 months due to the possible development of retroperitoneal and lung fibrosis [288]. It is not available in Italy. *Pizotifen* (1–4 mg/day), in a dated double-blind study, showed a certain efficacy in a limited number of patients with episodic CH (reduction >50 % of attack frequency in 36 % of the cases and interruption of cluster period in 21 %) [289]. *Lisuride* has been administered at variable dosages from 0.075 to 0.400 mg/day to patients with episodic and chronic cluster headache in an open study. In all patients the drug induced a benefit without relevant side effects [290]. *Lisuride* is available in Italy at the dosages of 0.2–0.5 mg and does not have the indication for CH.

#### Antiepileptics

A positive effect of *sodium valproate* (1–2 g/day) had been suggested by an open study but was not confirmed by a more recent RCT [291, 292]. In the latter study, drug/placebo was administered for 2 weeks and 50 % of responders were identified in the valproate group versus 62 % of placebo group [292]. Two open studies showed an effect of *topiramate* at different dosages (50 and 125 mg/day and 25–200 mg/day, respectively) with a remission within about 3 weeks from the beginning and a reduction of duration of the cluster period; a RCT (50–250 mg/day) did not show a superiority of topiramate compared with placebo [293–295]. After obtaining promising results in a single case, *gabapentin* has been tested in three open trials involving a limited number of patients at doses ranging from 800 to 3,600 mg/day. The drug, administered for 4–6 months, reduced significantly the frequency and intensity of attacks or interrupted the cluster period in at least 50 % of cases and was well tolerated [296–299]. Most of the antiepileptics listed above do not have the indication for CH in Italy.

*Ergotamine derivatives* i.m. *ergotamine* has been tested in an open study involving a limited number of patients during the active phase at dosages ranging from 0.25 to 0.50 mg/day to 0.25 mg four times per day. It induced a complete disappearance of attacks in all the patients without a reduction of cluster period. Side effects were somnolence, anorexia, bad taste and daze [300]. *Dihydroergotamine* i.v. was evaluated in two retrospective studies involving hospitalized patients. In the first study dihydroergotamine at the dosage of 0.5 mg three times per day interrupted the cluster period in patients with both episodic and chronic forms, refractory to other preventive treatments. In the second study dihydroergotamine i.v. (0.5 + 1 mg) and dihydroergotamine nasal spray 1 mg or dihydroergotamine s.c. at the dosage of 0.5–1 mg induced the disappearance of attacks or a reduction >50 % of attacks in 88 % of patients with episodic and 57 % of patients with chronic CH [301, 302]. Side effects were mild and only a few patients had to discontinue the drug.

#### Histamine

After the first observations of Horton (838), one dated open study involving few patients demonstrated that histamine sulphate i.v. (2.75 mg in 250 ml the first day, followed by 11 mg in 500 ml saline in the subsequent 9 days) induced a reduction of 75–100 % in one-third of cases, of 10–49 % in an another one-third of cases and no effect in the remaining cases [303].

#### Triptans

They were proposed for the short-term prophylaxis at the beginning of the cluster period instead of steroids (defined also as transitional therapy) or for the short-term prophylaxis of mini cluster. A reduction of the attacks was observed for *frovatriptan* (5 mg/day) in studies involving episodic CH patients treated with *verapamil* (retrospective study) and chronic CH patients resistant to preventive treatments (open study) [304]. In an open study *eletriptan* (40 mg two times per day for 6 days) induced 50 % reduction of attacks in one-third of the cases [305]. Responders were all treated with verapamil, whereas non-responders did not use preventive drugs. In a randomized double-blind versus placebo study sumatriptan 100 mg administered three times per day for 7 days did not induce a significant reduction of attack frequency [306].

#### Capsaicin

A first study demonstrated that the bilateral application of 300 μg per nostril, repeated to reach a complete desensitization, induced a significant reduction and disappearance of the attacks, while a second study demonstrated that the application in the ipsilateral nostril is equally efficacious, whereas the application in the contralateral nostril was ineffective [307, 308]. This effect was observed in the majority of patients with both episodic and chronic CH after 10 days of treatment. In the chronic patients after 25–40 days from the last application of capsaicin the attacks reappeared. Capsaicin is not available in Italy.

#### Melatonin

Its efficacy at the dosage of 10 mg per os has been tested in a limited number of patients with episodic form demonstrating the ability of the drug to reduce significantly the attack frequency. In responders this effect was obtained in 3–5 days [309, 310]. These promising results were not confirmed in a further pilot study involving patients with episodic CH [311].

Levels of recommendation for symptomatic and preventive drugs are reported in Table 13.

**Table 13:** Levels of recommendation for symptomatic (a) and preventive (b) treatment of cluster headache

Drug	Dosage	Level of recommendation	Comments	
(a) Symptomatic treatments				
Sumatriptan	6 mg s.c	I		
Sumatriptan	20 mg nasal spray	II	It is not approved by regulatory agency for cluster headache in Italy	
Zolmitriptan	5–10 mg nasal spray	II	It is approved by regulatory agency for cluster headache in Italy	
Oxygen inhalation	6–15 l/min for 15 min	I		

Drug	Dosage	Level of recommendation		Comments
		Episodic	Chronic	
(b) Preventive treatments for episodic and chronic cluster headache				
Verapamil	80–120 mg × 3 per day per os	I	IIIa	It is not approved by regulatory agency for cluster headache in Italy
Prednisone	50–75 mg/day per os for 3–7 days then gradually decreased to stop within 10 days	II	IIIb	It is not approved by regulatory agency for cluster headache in Italy Repeated use may, over time, induce severe adverse events
Pizotifen	Start with the dosage of 1 mg/day per os, increase the dosage to a maximum of 2.5 mg, to be reached in 2 weeks	IIIa	–	
Intranasal capsaicin	300 μg/day in the ipsilateral nostril repeatedly to obtain a complete desensitization	IIIa	IIIa	It is not available in Italy
Methysergide	Start with the dosage of 2 mg/day per os in three administrations, gradually increase the dosage (every 3–7 days) to the dosage of 8 mg/day. Maximum 6 month treatment	IIIb	IIIb	It is not available in Italy
Histamine sulphate	i.v. diluted in saline or 5 %: 1st day: 2.75 mg in 250 ml, 2nd to 10th day: 11 mg in 500 cc Starting flow rate of 10 ml/h, then 120 ml/h	–	IIIa	It is not available in Italy
Lithium carbonate	300 mg × 3 per day for no more than 22 weeks	–	IIIb	

Drugs with levels of recommendation IV include serotonin antagonists, ergotamine derivatives, triptans and melatonin

## Other trigeminal autonomic cephalgias (TACs)

### Paroxysmal hemicrania

Due to the low prevalence of this form, few studies have been carried out regarding the treatment of paroxysmal hemicrania (PH).

They are, in general, non-standardized, open, non-controlled studies and at times the studies lack relevant clinical information data, such as the effective duration of the treatment, the dosage of tested drugs and the patient follow-up.

PH, by definition, is a headache responsive to indomethacin and therefore, the diagnosis should be reconsidered in patients not responding to this drug at effective dosages (200 mg) [312–314].

Other drugs have been tested in PH patients who do not tolerate indomethacin. They include verapamil, COX-2 selective inhibitors (rofecoxib, colecoxib) and piroxicam [315–320].

## Short-lasting unilateral neuralgiform headache with conjunctival injection and tearing (SUNCT)

This rare headache form is also included in the TACs group and is therefore difficult to carry out controlled randomized clinical studies on its treatment [246].

Available data for several drugs have been obtained from case reports with limited patient series and rarely from observational studies.

In particular, studies involving lidocaine i.v. have been carried out on a few patients, whereas only case reports are available using i.v. or oral steroids [321].

Studies with more conspicuous patient series concern antiepileptic drugs used for preventive treatment. They include carbamazepine, gabapentin, topiramate and lamotrigine [321]. The most studied antiepileptic drug is lamotrigine due to its good efficacy and discrete tolerability.

### Levels of recommendation

It was impossible to define levels of recommendation for all drugs used for preventive treatment of PH and SUNCT because of the limited number of patients tested.

For both headache forms, levels of evidence, scientific strength of evidence, clinical effectiveness and side effects are reported in Tables 14 and 15.

**Table 14:** Level of evidence, scientific strength of evidence, clinical effectiveness, adverse events of preventive drug for Paroxysmal Headache

Drug	Daily dosage (mg)	Level of evidence	Scientific strength of evidence^a^	Clinical effectiveness	Adverse events	No. of cases	References
Indomethacin	25–50	C	0/+	+	–	1	[322]
Indomethacin	75	C	0/+	+++	Occasional, not severe	8	[323–330]
Indomethacin	150	C	0/+	+++	–	2	[330, 331]
Indomethacin	200–225	C	0/+	++	Occasional, not severe	3	[331, 332]
Verapamil	480	C	0/+	?	–	1	[333]
Piroxicam-β-cyclodextrine	20–40	C	0/+	++	–	6	[334]
Rofecoxib	25	C	0/+	+++	–	1	[317]
Rofecoxib	50	C	0/+	+++	Occasional, not severe	2	[317, 318]
Celecoxib	400	C	0/+	+++	–	1	[319]
Verapamil	240–320	C	0/+	++	–	10	[320]

^a^The scientific strength of evidence has been indicated as 0 (inefficacy)/+because there is no comparison with placebo or one active agent but sometimes the efficacy of the tested drug has been demonstrated

**Table 15:** Level of evidence, scientific strength of evidence, clinical effectiveness, adverse events of preventive drugs for SUNCT

Drug	Daily dosage (mg)	Level of evidence	Scientific strength of evidence^a^	Clinical effectiveness	Adverse events	No. of cases	References
Topiramate oral	75	C	0/+	++	Mild	2	[333]
Topiramate oral	50	C	0/+	+++	–	1	[334]
Carbamazepine oral	200	C	0/+	++	–	1	[335]
Carbamazepine oral	400	C	0/+	+++	–	1	[336]
Carbamazepine oral	600–1,000	C	0/+	?	–	5	[337–340]
Carbamazepine oral	2,000	C	0/+	?	–	1	[341]
Gabapentin oral	800–2,700	C	0/+	+++	–	3	[342–344]
Lamotrigine oral	100–200	C	0/+	++	–	12	[345–350]
Lamotrigine oral	300–400	C	0/+	+++	–	2	[351, 352]
Verapamil	480	C	0/+	?	–	1	[353]
Topiramate oral	200	C	0/+	+++	–	1	[354]
Methylprednisolone oral	≤1 /kg	C	0/+	+++	–	3	[355]
Oxcarbazepine and gabapentin oral	600/400	C	0/+	++	Mild	1	[356]
Lidocaine i.v.	1.3 /kg/h	C	0/+	+++	–	1	[357]

^a^The scientific strength of evidence has been indicated as 0 (inefficacy)/+ because there is no comparison with placebo or one active agent but sometimes the efficacy of the tested drug has been demonstrated

## Primary headaches management in particular conditions

### Emergency Department

In the Emergency Department (ED) acute treatment must be simple, based on a few drugs with a clear evidence of efficacy, administrable through rapid absorption routes (rectal, intramuscular or endovenous) and rapidly effective [358].

### Migraine

Among NSAIDs, ketorolac 60 mg, administered intramuscularly, followed by a subsequent dose of 30 mg after 8 h, has been shown to be more effective than intranasal sumatriptan, but less effective than phenotiazines in relieving migraine attacks in the ED [67, 68, 359].

Another first-choice treatment is ASA 1,000 mg with or without metoclopramide [360]. Metamizole has been shown to be significantly more effective than placebo. Its potential side effects should be taken into account including severe hypotension, agranulocytosis and allergic reactions [361].

Positive data are available for dihydroergotamine 2 mg nasal spray or suppository but the drug is not available in Italy and is less effective than sumatriptan and phenotiazine [71].

Subcutaneous, intranasal and rectal sumatriptan should be available in the ED and it may be particularly useful in migraine patients with nausea and/or vomiting [362–364].

Some evidence on the efficacy of 1 mg of magnesium sulphate by intravenous route in the treatment of migraine attacks in the ED needs to be confirmed [365].

In the presence of nausea and vomiting 10 mg metoclopramide by intramuscular route can be useful, even if occasional adverse events with this drug should be considered including sedation, akathisia, acute dystonic crises and other extrapyramidal symptoms such as stiff neck and oculogyric crises [84].

Among the other antiemetics, ondansetron 4–8 mg by intravenous route may be used.

Prochlorperazine has been shown to be more effective than placebo, metoclopramide and other agents in the symptomatic treatment of migraine attacks in the ED [366, 367]. In association with diphenhydramine, prochlorperazine has been shown to be more effective than sumatriptan and sodium valproate i.v. [363, 368]. Its most frequent adverse event is sedation.

Meperidine, an opioid analgesic, although effective, may cause frequent adverse events such as sedation, disorientation, akathisia, gastrointestinal disturbances [369]. Furthermore, it can induce addiction and dependence and therefore it is not recommended by the group of experts.

Benzodiazepines (especially diazepam 5–10 mg, administered by i.v.) are useful in case of concomitant anxiety.

In the status migrainosus and in the treatment of attack recurrence, dexamethasone 10 mg followed by a subsequent dose of 4 mg every 6 h can be used, even if there are no consistent data on its efficacy in these two conditions [104, 107, 370–373].

### Tension-type headache

The group of experts suggests the use of NSAIDs, i.m. or i.v., to obtain a more significant and rapid relief of pain in the rare cases in which tension-type headache patients present to the ED.

Oral benzodiazepines can also be useful in the case of attacks of great intensity, particularly in patients with concomitant anxiety.

Metoclopramide i.v. has demonstrated a modest analgesic efficacy at the dosage of 10 mg [374].

The efficacy of chlorpromazine i.v., at the dosage of 10 mg, in tension-type headache patients presenting to the ED has also been demonstrated [375]. The most frequent adverse event is sedation, occasionally extrapyramidal symptoms or akathisia may occur.

### Cluster headache

Cluster headache attack, given its short duration, is rarely treated in the ED. However, CH patients with frequent daily attacks can refer to the ED. In these cases indications for an adequate prophylaxis therapy should be provided and patients should be addressed to a Headache Center, possibly in the same hospital, to set up the most suitable preventive treatment.

## Headache management in pregnancy and lactation

### Migraine

No studies are available on the use of both symptomatic and prophylactic drugs in pregnant women. There are also few data on the risk related to their use during pregnancy, delivery and breast-feeding. Available recommendations come from the Regulatory Agencies of the single countries [376, 377].

It is worth noting the potential teratogenic risk of methysergide, other ergot alkaloids and metoclopramide, the use of which is unadvisable during pregnancy and breast-feeding.

There are conflicting data on the potential risk of foetal malformations, although rare, for amitriptyline. In the case of its use during pregnancy, amitriptyline should be suspended, at least 7 days before delivery to reduce the likelihood of a neonatal withdrawal syndrome which is characterized by respiratory distress and trouble feeding. Amitriptyline should be avoided during lactation [378].

According to the group of experts, the majority of symptomatic and prophylactic drugs are not recommended during pregnancy [379, 380]. For symptomatic treatment, the first-choice drug is paracetamol, particularly during the first trimester of pregnancy. NSAIDs use, if needed, should be limited to second and third trimester of pregnancy [381]. Use of triptans is unadvisable during pregnancy. Data from pregnancy registries show a higher premature delivery frequency and lower weights for newborns in comparison with the average (sumatriptan), but no severe adverse events or complications during delivery (sumatriptan, rizatriptan) [54, 382–392].

In the case of nausea or vomiting during attacks, domperidone is the best choice drug. Antiemetics and sedative phenotiazinic drugs’ use is not recommended [393, 394].

Prophylactic drugs should not be used, or at least only rarely, or at least used only rarely, during pregnancy, with particular regard to antiepileptics due to their teratogenic potential [395–397]. The only exceptions are magnesium and beta-blockers (propranolol, metoprolol in the second and third trimesters of pregnancy) for which there is no evidence of teratogenicity (level of recommendation II for both). Beta-blockers could have toxic effects on the newborn and may be responsible for intrauterine growth retardation, hypoglycemia, bradycardia and respiratory depression [398]. They are not recommended in women who present with migraine with aura during pregnancy; they can be used, on the contrary, during lactation. In unresponsive cases, dexamethasone or prednisone may be used [399].

### Tension-type headache

There are no studies on TTH during pregnancy. Paracetamol should be preferred for the attack treatment. If preventive treatment is needed, non-pharmacological therapy option should be preferred.

### Cluster headache

Pregnant women suffering from CH [400, 401] should be informed on the treatment benefits and risks and on the drug potential teratogenic effect. Dosage and number of administrations should be reduced to the minimum. Treatments to be preferred are oxygen, prednisone and verapamil. The use of subcutaneous or intranasal sumatriptan should be limited as much as possible. If verapamil cannot be used, gabapentin should be preferred as prophylactic treatment. During lactation, oxygen and sumatriptan may be used as symptomatic drugs and prednisone/prednisolone, verapamil and lithium for prophylaxis.

## Headache management in the elderly

In several guidelines for primary headaches specific indications for the management of elderly headache patients are lacking. Furthermore, specific controlled studies are lacking and therefore recommendations are mainly based on opinions of experts.

## Migraine

Among migraine symptomatic drugs, the safest is paracetamol. NSAIDs may also be used, in absence of contraindications. They have however potentially severe adverse events, particularly gastroenteric and renal side effects [399].

Among antiemetics domperidone should be preferred to metoclopramide which, particularly in the elderly, can be responsible for extrapyramidal side effects.

Controlled studies on the use of triptans by patients over 65 years are lacking. From the few data available in the literature, the use of triptans in a general population did not demonstrate a greater frequency of cardio- and cerebrovascular complications in patients over 65 years without vascular risk factors (1,037). In the experts’ opinion their use is therefore possible in the presence of low frequency of attacks, after a careful evaluation of cardio- and cerebrovascular risks in the single patient. Conversely, ergot derivatives are contraindicated due to their widespread vasoconstrictive action. Use of combination drugs must be limited due to the risk of abuse. The use of opioid drugs is unadvisable because of the adverse events, such as disorientation, sedation and nausea [400].

First-choice drugs for prophylaxis are beta-blockers, particularly atenolol, metoprolol and bisoprolol because of their good tolerability profile [401]. Their contraindications are chronic obstructive pulmonary disease, bradycardia or ventricular hyperkinetic arrhythmias and depression. Beta-blockers must be used with caution in presence of diabetes. The use of flunarizine should be limited because of its potential side effects with particular regard to extrapyramidal disturbance and parkinsonism.

In relation to the high prevalence of depression in the elderly, antidepressants represent a good therapeutic option [402]. Although effective, tricyclic antidepressants (amitriptyline, nortriptyline) are not free from adverse events, which may be even severe, particularly in the elderly. They include sedation, cognitive disturbances, cardiac rhythm disturbances (tachycardia, hyperkinetic arrhythmias), postural hypotension, acute glaucoma and urinary retention, particularly in patients with prostatic hypertrophy [403].

SSRI and SNRI are better tolerated but they have not shown a significant efficacy for migraine prophylaxis in randomized controlled studies. Their usefulness must be evaluated in individual cases.

The use of antiepileptics should be limited to migraine with a high frequency of attacks and in the case of comorbidity of migraine with epilepsy. Topiramate and sodium valproate should be preferred based on the greater availability of data showing efficacy, but they are not free from adverse events. In particular, topiramate may induce cognitive disturbances and sedation and, rarely, is responsible for visual disturbances (acute myopia onset, glaucoma) and nephrolithiasis. Topiramate use is contraindicated in the last two disturbances [403].

Sodium valproate, conversely, may cause tremors, ataxia and hepatotoxicity (particularly in patients with previous hepatic disturbances). Other antiepileptics with lesser evidence of efficacy, but a higher tolerability profile can be alternatively used, such as gabapentin and, in some cases, pregabalin [401].

Given the recent evidence of efficacy of lisinopril and candesartan in migraine prophylaxis, their use can be considered because of the good tolerability profile especially in the presence of hypertension.

### Tension-type headache

There are no studies regarding the treatment of tension-type headache in elderly patients. Paracetamol and NSAIDs are recommended for symptomatic treatment, keeping in mind the considerations already reported for migraine.

Given the frequent comorbidity with depressive disturbances, antidepressants are the first-choice drugs among preventive drugs. Mirtazapine, SSRI (fluoxetine, paroxetine, fluvoxamine, sertraline, citalopram and escitalopram) or SNRI (venlafaxine, duloxetine) should be preferred because of their greater tolerability profile in comparison with tricyclics, even if controlled and randomized studies of elderly tension-type headache are lacking.

Drugs with fewer evidence of efficacy are tizanidine and onabotulinumtoxinA for which, in the literature, data are contrasting and, in the majority of cases, negative.

Non-pharmacological procedures are often the only applicable treatments although there is no evidence of their efficacy in geriatric patients. It should be emphasized that the psychiatric approach, if needed, is particularly difficult in elderly patient.

### Cluster headache

Sumatriptan s.c. is not recommended because of the limitation in its use by patients over 65 years of age and for the possible occurrence of angina and hypertensive crises. The first-choice drug is oxygen 100 %, by inhalation using a mask, at the rate of 7 l/min for 15 min. For preventive treatment the best drug for geriatric patients is verapamil which is well tolerated by patients up to a dosage of 480 mg/day. Side effects for which its suspension is recommended are rare (bradycardia, hypotension, constipation, peripheral edemas). It can be useful to associate melatonine with verapamil, at an evening dosage of 6–10 mg [402, 403]. In the case of an unsatisfactory response to verapamil, the second choice drug is prednisone at the dosage of 50 mg, in association with a proton-pump inhibitor. Its use should be limited to a short period (7–14 days). The use of lithium must be restricted to cases refractory to the other prophylactic treatments given the low tolerability in the elderly. The periodic plasma monitoring of lithium levels is needed and values should be maintained within the therapeutic range (0.4–1.2 mEq/l).

There are no data regarding the use of sodium valproate and topiramate by CH elderly patients in the unresponsive chronic forms.

As far as the non-pharmacological therapies are concerned, there are no data relative to the application in the elderly of new procedures such as the hypothalamic deep brain stimulation and greater occipital nerve stimulation.

With regard to the treatment of the other primary headaches in the elderly, such as hypnic headache and paroxysmal headache, one can refer to the papers recently published by our subgroups of experts on other primary headaches (2–5).

## Non-pharmacological therapy of primary headaches

Just as for the pharmacological therapy the non-pharmacological therapy includes a symptomatic and a preventive treatment. The non-pharmacological techniques can be a valid alternative or complementary treatment. They are particularly indicated in the case of pregnancy, breast feeding, multiple therapies for comorbid diseases, poor tolerability of drugs, childhood and elderly.

### Acute attack treatment

Most of the evidence for the techniques used in controlling headache attacks is purely anecdotic because RCTs are lacking. A study on 400 primary headache (including migraine, TTH and CH) patients showed the scarce and momentary efficacy of self-administered manoeuvres on various regions of the head (compression, application of cold or heat, massage). In spite of this, 46 % of the subjects used the manoeuvres constantly, at each attack [404].

In migraine a study compared the efficacy of sumatriptan with acupuncture showing a similar efficacy of the two drugs but it should be pointed out that it is difficult to apply acupuncture at the moment of attack [405].

Transcranial magnetic stimulation (TMS) determined a reduction or the remission of pain in 69 % of migraine with aura patients versus 48 % in the group treated with sham technique (level of recommendation II) [406]. Some evidence is available for the therapeutic blockade of greater occipital and supraorbital nerves in migraine patients [407].

### Preventive treatment

Biofeedback is the non-pharmacological technique of first choice for migraine prophylaxis (level of recommendation I) [408]. The effect of biofeedback is slower but more lasting when compared with drug therapy. The association of biofeedback and pharmacological therapy induces a more marked effect [409]. Conflicting results deriving from the comparison with sham-acupuncture were obtained for acupuncture (level of recommendation II). However, in a recent study, its efficacy was found to be similar to that of flunarizine [410]. Acupuncture is contraindicated in the presence of coagulation deficits, cardiac pacemaker, cardiac arrhythmias and epilepsy. The expertise of the operator is fundamental. Needles have to be sterilized to avoid infections. Other non-pharmacological therapies, including relaxing techniques, sleep therapies, spinal manipulations, stretching, mobilization, physiotherapy and TMS, were rated at the level of recommendation III for migraine preventive therapy prophylaxis on the base of the available scientific evidence.

Further studies with homogeneous methods are needed to definitively demonstrate the efficacy of TENS, physical activity, anaesthetic blockade of the greater occipital and supraorbital nerves, diet restriction and orthodontic/gnathologic techniques (all of these were rated at level of recommendation IV). Several retrospective studies and three prospective studies (one of these was double-blind placebo-controlled) monitoring the post operative course after patent foramen ovale closure for cardiovascular indications showed improvement of concomitant migraine (level of recommendation III) [411, 412]. Promising results come from the surgical deafferentation of muscle trigger points in frontal, temporal and parietal regions (level of recommendation IV).

Basing upon the current scientific evidence biofeedback and acupuncture are the first-choice non pharmacologic therapies for the prophylaxis of tension-type headache. Biofeedback shows a slower but more lasting therapeutic effect than amitriptyline [409]. This technique induces a greater efficacy when it is associated with other non pharmacologic or pharmacologic options (level of recommendation I) [413]. Acupuncture was found to be more effective than drug preventive therapy and sham acupuncture and as effective as other therapies (physiotherapy, relaxation programs and massage + relaxation program) in a Cochrane review of 11 studies [414]. For contraindications and limits of this technique, see above.

Many other non pharmacological treatments are useful in the prevention of TTH, although further well-conducted studies are needed to support their efficacy. They include cognitive-behavioural therapy (as effective as amitriptyline but slower) [415], short-term psychodynamic psychotherapy (determining a lower rate of relapse in analgesic overuse than placebo after weaning) [416], osteopathic, chiropractic and massage protocols (contraindicated in the case of fractures, bone lesions and osteoporosis) [417, 418], Physiotherapy/Physical Exercise (several techniques showed to be effective but the results are not comparable because non homogeneous methods have been used in different studies) [419, 420] and orthodontic/gnathological techniques (an intraoral removable device induced an improvement similar to amitriptyline’s one) [421]. All the aforementioned non pharmacologic techniques have been rated at the level of recommendation III. Moreover, the transcutaneous electrical nerve stimulation (TENS) is largely used in clinical practice to alleviate chronic pain but no evidence is up to now available to demonstrate its efficacy in TTH (level of Recommendation IV) [422].

Deep brain stimulation with stimulator implantation in the posterior hypothalamus represents the most innovative surgical technique for aborting chronic refractory cluster headache. Pain relief has been obtained in 83 % of 16 cluster headache patients followed up for 5 years, with 61 % of patients completely free from pain [423]. The efficacy of this approach has been confirmed in nine cluster headache patients followed up for 2 years in 71 % of cases [424]. In a recent double-blind, prospective, crossover study versus sham stimulation involving 11 cluster headache patients a reduction of attacks >50 % was observed in six patients [425]. This technique is not free from risks: a case of death for cerebral haemorrhage was reported in a case series of seven cluster headache patients who underwent hypothalamic deep brain stimulation [426]. It should be reserved only to chronic cluster headache patients, totally resistant to all therapeutic strategies (level of recommendation II).

### Levels of recommendation

Levels of evidence, scientific strength of evidence and clinical effectiveness for symptomatic and preventive non-pharmacological treatments are reported in Tables 16 and 17 for migraine, Tables 18 and 19 for tension-type headache and Tables 20 and Table 21 for cluster headache.

**Table 16:** Symptomatic non-pharmacological treatments for migraine

Treatment	Level of evidence	Scientific strength of evidence	Clinical effectiveness	Adverse events	Level of recommendation	References
Pain relieving manoeuvres	–	–	0	–	IV	[404]
Acupuncture	–	–	+	–	IV	[405]
TMS	B	++	+	–	II	[406, 427, 428]
Mechanical compression	–	–	0	–	IV	[429]
GON blockade	C	++	+	–	III	[430]

*TMS* transcranial magnetic stimulation, *GON* greater occipital nerve

**Table 17:** Preventive non-pharmacological treatments for migraine

Treatment	Level of evidence	Scientific strength of evidence	Clinical effectiveness	Adverse events	Level of recommendation	References
Biofeedback	A	++	++	–	I	[408, 409, 431, 432]
Relaxation	C	0/+	+	–	III	[433, 434]
Cognitive-behavioural treatment	–	–	+	–	IV	[435–437]
Sleep	C	++	+	–	III	[429, 438]
Chiropractic osteopathy	C	0/+	+	–	III	[419, 422, 439–441]
Physiotherapy	C	+	+	–	III	[442–444]
Acupuncture	A	++	+	Rare	II	[445]
Transcutaneous electrical nerve stimulation (TENS)	–	–	?	–	IV	[442]
Transcranial magnetic stimulation (TMS)	C	+	+	Rare	III	[428, 446]
Physical activity	–	–	?	–	IV	[447]
Anaesthetic blockade	C	?	?	Rare	IV	[407]
Diet	–	–	?	–	IV	[448]
Orthodontic and gnathological techniques	C	+	+	–	IV	[422, 449]
PFO Closure	C	++	?	Rare, severe	III	[411, 412]
Occipital nerve stimulation	C	–	?	Rare		[450]
Trigger points deafferentation	–	–	?	–	IV	[451]

*PFO* patent foramen ovale closure

**Table 18:** Symptomatic non-pharmacological treatments for tension-type headache

Treatment	Level of evidence	Scientific strength of evidence	Clinical effectiveness	Adverse events	Level of recommendation	Reference
Pain relieving manoeuvres	–	–	0	–	IV	[404]

**Table 19:** Preventive non-pharmacological treatments for tension-type headache

Treatment	Level of evidence	Scientific strength of evidence	Clinical effectiveness	Adverse events	Level of recommendation	References
Biofeedback	A	++	++	–	I	[408, 413, 431]
Cognitive-behavioural treatment	–	–	+	–	IV	[416, 435, 437, 452]
Strategic short-term psychotherapy	–	–	+	–	IV	[417]
Chiropractic osteopathy	C	+	+	–	III	[418, 419]
Physiotherapy	C	+	+	–	III	[419, 427, 453–458]
Acupuncture	A	++	+	Rare	II	[452]
Transcutaneous electrical nerve stimulation (TENS)	–	–	?	–	IV	[422]
Physical activity	–	–	?	–	IV	[421]
Pranotherapy	–	–	?	–	IV	[459]
Orthodontic and gnathological techniques	C	+	+	–	IV	[414, 422, 449, 460]

**Table 20:** Symptomatic non-pharmacological treatments for cluster headache

Treatment	Level of evidence	Scientific strength of evidence	Clinical effectiveness	Adverse events	Level of recommendation	Reference
Pain relieving manoeuvres	–	–	0	–	IV	[404]

**Table 21:** Preventive non-pharmacological treatments for cluster headache

Treatment	Level of evidence	Scientific strength of evidence	Clinical effectiveness	Adverse events	Level of recommendation	References
Anesthetic blockade	−	−	?	Rare	IV	[461]
Lesion of trigeminal nucleus	C	+	?	Rare, severe	IV	[462–467]
Occipital nerve stimulation	C	−	?	Rare	IV	[468, 469]
Hypotalamic deep brain stimulation	B	++	+++	Rare, very severe	II (only for chronic refractory CH)	[423, 470, 471]

## Members of the Ad Hoc Committee

Marta Allena

Vincenzo Amenta

Giuseppe Capo

Stefano Caproni

Claudio Antonio Caputi

Cinzia Cavestro

Biagio Ciccone

Cecilia Condello

Fernando Conte

Ilenia Corbelli

Alfredo Costa

Alfio D’Agati

Giovanni D’Antonio

Filippo Dainese

Giorgio Dalla Volta

Emilio De Caro

Francesco De Cesaris

Milena De Marinis

Flavio Devetag

Graziella Di Meo

Franco Di Palma

Girolamo Di Trapani

Vincenzo Firetto

Enrico Ferrante

Andrea Giorgetti

Sara Gori

Enrico Grappiolo

Filippo Lanaia

Giovanni Battista La Pegna

Carlo Lisotto

Ferdinando Maggioni

Federico Mainardi

Annamaria Miccoli

Franco Mongini

Cesare Morandi

Gianni Moro

Marco Mucchiut

Antonio Palmieri

Patrizia Panicucci

Giuliano Relja

Paolo Rossi

Eugenia Rota

Francesco Salvadori

Mariantonietta Savarese

Vittorio Sciruicchio

Lucia Testa

Marco Trucco

## Lay Members

Angela Piperni

Barbara Radaelli

Gianfranco Tazzioli
